# Soliloquy

**Published:** 1885-11-20

**Authors:** 


☞ SOLILOQUY.—By Jove! Let me think. I have not paid my subscription since—well, I don't know how long. Several times the Editor has politely invited my attention to the matter, editorially, and he has even gone to the trouble and expense of mailing me a circular, two or three times. Now that I come to think of it, I have really treated him badly. I know it is a great deal of trouble and expense to publish and mail these journals every month, year in and year out, as he has so patiently and faithfully done. The journal has been to me a source of pleasure, and has furnished me a great deal of useful information. "*I am really ashamed of myself*," and will go *right now* and remit my dues to the RECORD. But hold; I haven't got the money! No matter. I will get it some how. I can borrow or collect it, if I try. Only a single visit to a patient pays for it How cheap for twelve months' of valuable reading! Shame on me!

